# Intrastrand Photo-Crosslinking of 5-Fluoro-2′-O-methyl-4-thiouridine-Modified Oligonucleotides and Its Implication for Fluorescence-Based Detection of DNA Sequences

**DOI:** 10.1021/acs.joc.4c01597

**Published:** 2024-10-24

**Authors:** Joanna Nowak-Karnowska, Katarzyna Taras-Goslinska, Shozeb Haider, Bohdan Skalski

**Affiliations:** † Department of Chemistry, Adam Mickiewicz University, Poznań 61-614, Poland; ‡ School of Pharmacy, University College London, London WC1N 1AX, U.K.; § Center for Advanced Technology, Adam Mickiewicz University, Poznań 61-614, Poland

## Abstract

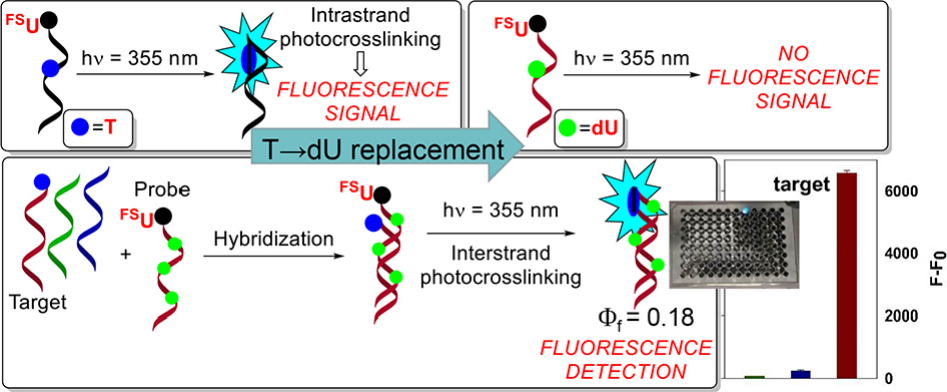

DNA photo-crosslinking reactions occur widely in biological systems and are often used as valuable tools in molecular biology. In this article, we demonstrate the application of an oligonucleotide 5-fluoro-2′-O-methyl-4-thiouridine (**^FS^U**)-containing probe for the fluorescent detection of specific DNA sequences. The design of the probe was predicated on studies of agents that could adversely affect its efficiency. The most important of these is the intrastrand photo-crosslinking of single-stranded oligodeoxynucleotides bearing ^**FS**^**U**. Our research findings indicate that ^**FS**^**U** after photoexcitation can react with nonadjacent bases; specifically, it can react with distant thymine and cytosine residues in the chain, forming fluorescent and nonfluorescent intrastrand crosslinks, respectively. In addition, partial photooxidation of the ^**FS**^**U** residue to 5-fluorouridine was also observed. The results of the study are significant in terms of the use of ^**FS**^**U**-labeled oligonucleotide probes in the fluorescence-based detection of specific DNA sequences because the creation of a fluorescent intrastrand crosslink could produce a false signal. To overcome this problem, replacing thymidine with deoxyuridine in the ^**FS**^**U**-labeled oligonucleotide probe is proposed and tested.

## Introduction

Crosslinking of DNA can be induced by chemical reaction or exposure to UV irradiation.^1^ The formation of interstrand crosslinks in DNA causes the inhibition of transcription and replication^2^ and can modulate nucleic acid conformation,^3^ including aptamer,^4^ G-quadruplex,^5^ and i-motif structures.^6^ Short DNA oligonucleotides capable of creating crosslinks are valuable model systems in the study of DNA damage repair mechanisms.^7^ Furthermore, crosslinking may have potential applications for detecting specific DNA/RNA sequences^8^ and identifying targets for bioactive small molecules.^9^

Among the photoactivable crosslinking agents belonging to modified nucleobases, 4-thiouridine (^**4S**^**U**)^10^ and 6-thioguanosine (^**6S**^**G**)^11^ should be distinguished. We have previously described the UV-induced interstrand crosslinking of DNA duplexes labeled with 5-halogeno analogs of ^**4S**^**U**, namely, 5-fluoro-2′-O-methyl-4-thiouridine (^**FS**^**U**)^12^ and 5-chloro-2′-deoxy-4-thiouridine (^**ClS**^**dU**).^13^ Similar to the native ^**4S**^**U**, 5-fluoro-4-thiouridine and 5-chloro-4-thiouridine show high photoreactivity toward thymidine. However, unlike ^**4S**^**U**, which mostly forms 6–4 and 5–4 pyrimidine–pyrimidone adducts with thymidine,^14^ 5-fluoro- and 5-chloro-4-thiouridine undergo photo-cycloaddition with thymidine, producing a unique pair of diastereomeric, highly fluorescent, and thermally stable tricyclic adducts.^15^ It should be noted that 5**-**fluoro-4-thiouridine exhibits a photoreactivity much higher than that of the 5-chloroderivative. We have already demonstrated that the photoreaction occurs both under selective excitation of monomeric 5-fluoro-4-thiouridine in the presence of thymidine^15^ and in double-stranded oligonucleotides leading to fluorescent interstrand crosslink formation with nearly quantitative yield.^12,13^

**Figure 1:**
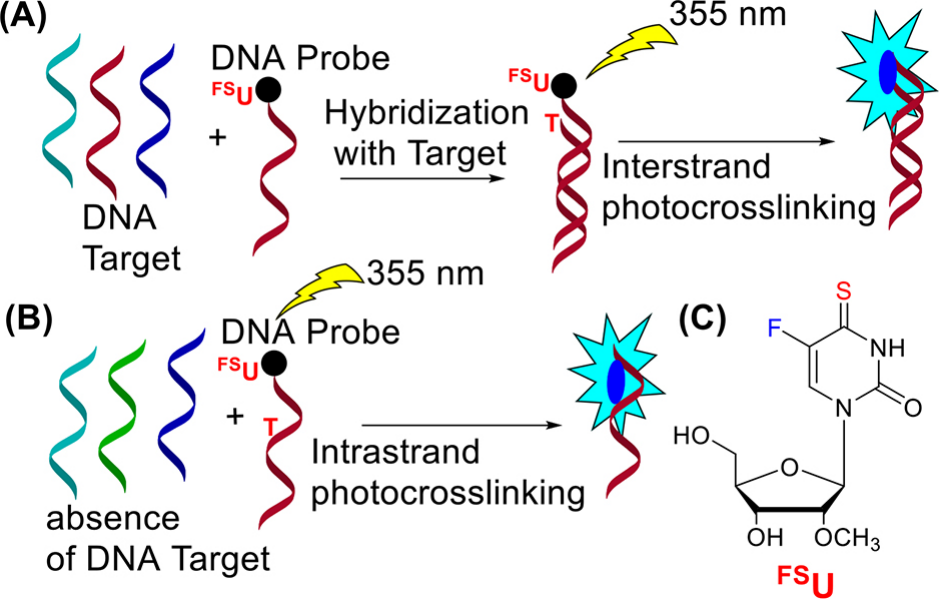
Fluorescence signal generation for interstrand photo-crosslinking (A) and intrastrand photo-crosslinking of 5-fluoro-2′-O-methyl-4-thiouridine (^**FS**^**U**) with **T** in DNA (B) and the structure of ^**FS**^**U** (C).

The formation of fluorescent crosslinks enables easy and quick monitoring of the reaction and has potential application for the rapid and fluorescence-based detection of specific DNA sequences (Figure 1A).

For application purposes, the DNA fluorescent probe sequence must bind specifically to the target DNA. In the case of interstrand DNA photo-crosslinking, the Watson–Crick base pairing and strand complementarity meet this requirement. However, in the absence of the target, there is a risk of competitive intrastrand photo-crosslinking reactions. Therefore, it is necessary to study the possibility of intrastrand crosslink formation, as it can lead to false detection signals (Figure 1B).

In this report, we present the results of the irradiation of synthetic oligonucleotides labeled with ^**FS**^**U**. Depending on the ODN sequence, the formation of intrastrand crosslinks between ^**FS**^**U** and **T** or **C** was observed. The influence of temperature of irradiated solutions on the distribution of photo-crosslinks and other photoproducts will also be discussed. The probe DNA sequence labeled with ^**FS**^**U** will also be tested for the detection of the pseudotarget from the DNA of the HPV-16 virus.

## Results and Discussion

Oligodeoxynucleotides modified with 5-fluoro-2′-O-methyl-4-thiouridine (^**FS**^**U**) (**ODN 1**: 5′ CGATACGA^**FS**^**U**A 3′; **ODN 2**: 5′ A^**FS**^**U**AGCATAGC 3′; **ODN 3**: 5′ ATAGCA^**FS**^**U**AGC 3′) were synthesized using “ultramild” DNA synthesis, as described previously.^16^**ODN 1–3** have the same nucleoside compositions. In **ODN 1**, ^**FS**^**U** is located near the 3′ end of the strand, while **ODN 2** is the reversed sequence of **ODN 1. ODN 3** has the same sequence as **ODN 2**, but the positions of ^**FS**^**U** and **T** are swapped.

**Figure 2:**
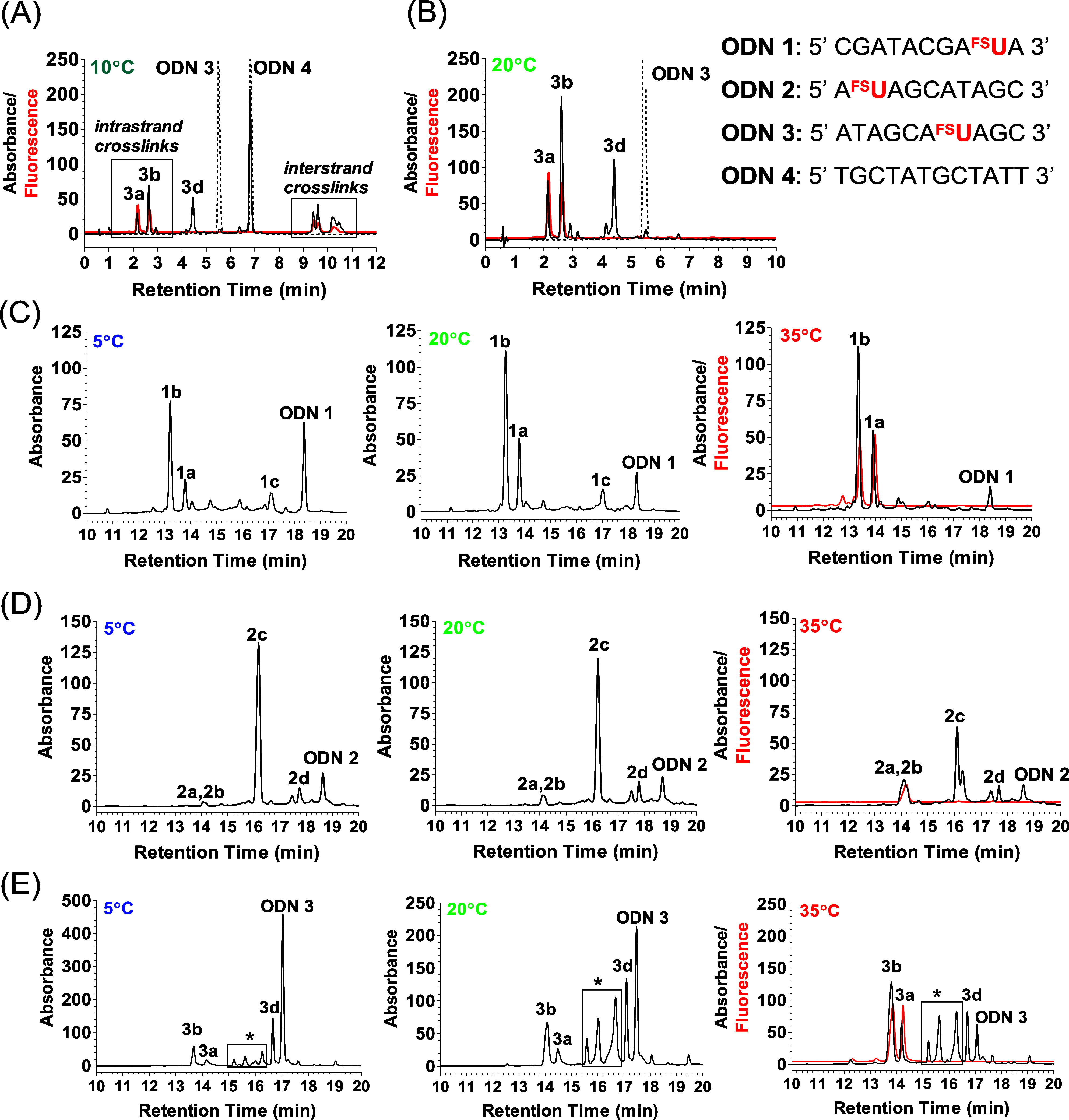
HPLC absorbance (at 260 nm, black lines) and fluorescence elution profiles (at 460 nm, λ_ex_ 370 nm, red lines) before (dotted line) and after (solid line) irradiation with 355 nm for 10 min of the **ODN 3**:**ODN 4** duplex at 10 °C (A), **ODN 3** at 20 °C (B), **ODN 1** (C), **ODN 2** (D), and **ODN 3** (E) after irradiation at 5, 20, and 35 °C for 150 s. (A, B) Waters XBridge OST C18 Column, 2.5 μm, 4.6 × 50 mm, 40 °C, eluted with 0.1 M TEAA, using a linear gradient of 7–10% acetonitrile over 10 min; flow rate: 1 mL/min. (C–E) Agilent Poroshell 120 EC-C18 Column, 2.7 μm, 4.6 × 150 mm, 40 °C, eluted with 0.1 M TEAA, using a linear gradient of 5–15% acetonitrile over 20 min; flow rate: 0.5 mL/min. * indicates intermediate photoproducts.

We have previously tested the photochemical reactivities of **ODN 1** and **ODN 2** in the presence of the complementary oligonucleotide and observed almost quantitative formation of interstrand photo-crosslinks between ^**FS**^**U** and **T**.^12^ We decided to perform an analogous experiment in the case of **ODN 3**. We irradiated **ODN 3** with λ = 355 nm in the presence of 1.1 mol equiv of **ODN 4**. While the total conversion of **ODN 3** was noticed, 66% of **ODN 4** (based on high-performance liquid chromatography (HPLC)) remained unreacted (Figure 2A).

**Figure 3:**
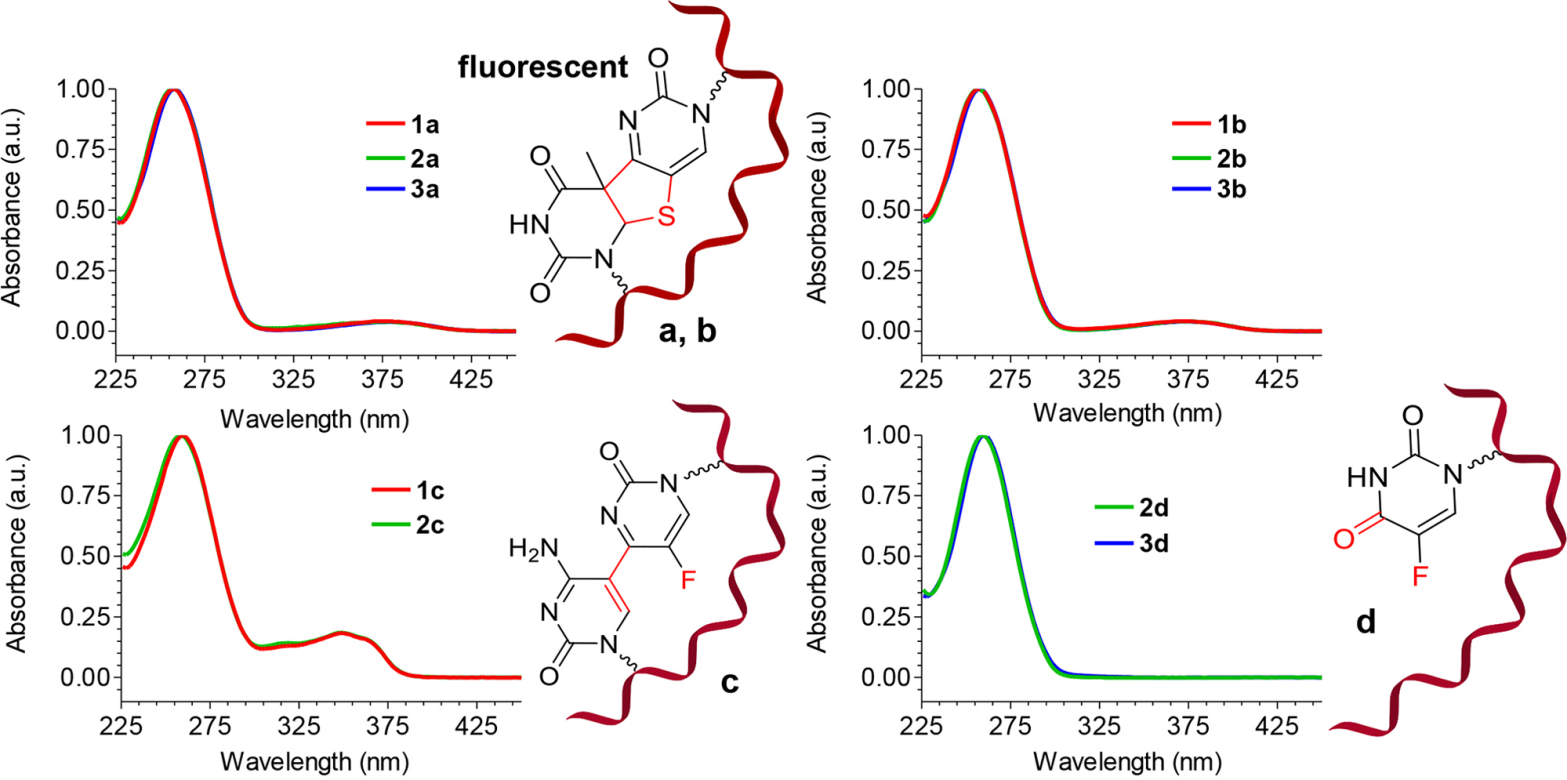
Structure and normalized absorption spectra of intrastrand photo-crosslinks of ^**FS**^**U** with **T** (**a**, **b**), photo-crosslinks of ^**FS**^**U** with **C** (**c**), and product of photooxidation of ^**FS**^**U** (**d**) formed in the studied oligonucleotides **ODN 1–3**.

Different from earlier reports, apart from the formation of fluorescent interstrand photo-crosslinks (see Figure S1 for MALDI-TOF MS spectra), we also observed fluorescent photoproducts with shorter retention times (Figure 2A). The shorter retention times of these photoproducts and their absorption and emission properties (higher ratio of the intensity of the absorption bands at 370 nm to the band at 260 nm than in the case of interstrand photo-crosslinks) prompted us to conclude that these are intrastrand photo-crosslinks. Furthermore, we observed the formation of intrastrand crosslinks during the irradiation of **ODN 3** alone (as shown in Figure 2B). Despite the irradiation of the mixture of **ODN 3** and **ODN 4** with varying excess of **ODN 4** (1–2 mol equiv) at 10 °C, i.e., well below the melting temperature of the duplex **ODN 3**:**ODN 4**, intrastrand photo-crosslinking of **ODN 3** was always observed. These reactions have not been observed before on short oligonucleotides, so the above findings have also guided us to further investigate the intrastrand photo-crosslinking of oligonucleotides **ODN 1–3.** Aqueous solutions of **ODN 1–3** in 0.1 M phosphate buffer, pH 7.0, were irradiated at 20 °C, and the progress of the reaction was monitored spectrophotometrically (Figure S2) and by HPLC (Figure 2C–E). Under these conditions (λ = 355 nm), selective excitation of ^**FS**^**U** was achieved. The difference in the reaction rate for **ODN**s **1–3** was observed (Figure S3), with the fastest conversion occurring for **ODN 1**. We also measured the quantum yields for the disappearance of **ODN**s **1–3** (Φ = 0.06, 0.045, and 0.02 for **ODN 1**, **ODN 2**, and **ODN 3**, respectively), reflecting the higher photoreactivity of **ODN 1** in relation to the others. For all oligonucleotides, after irradiation for 150 s, the formation of the same photoproducts (**a**–**d**) with appropriate nucleoside sequences **1**–**3** (corresponding to **ODN 1**–**3**) was observed but with different yields (Figure 2C–E). Since **ODN**s **1**–**3** have the same nucleoside composition, photoproducts (**a**–**d**) obtained from **ODN**s **1**–**3**, respectively, have similar UV absorption spectra (Figure 3).

**Figure 4:**
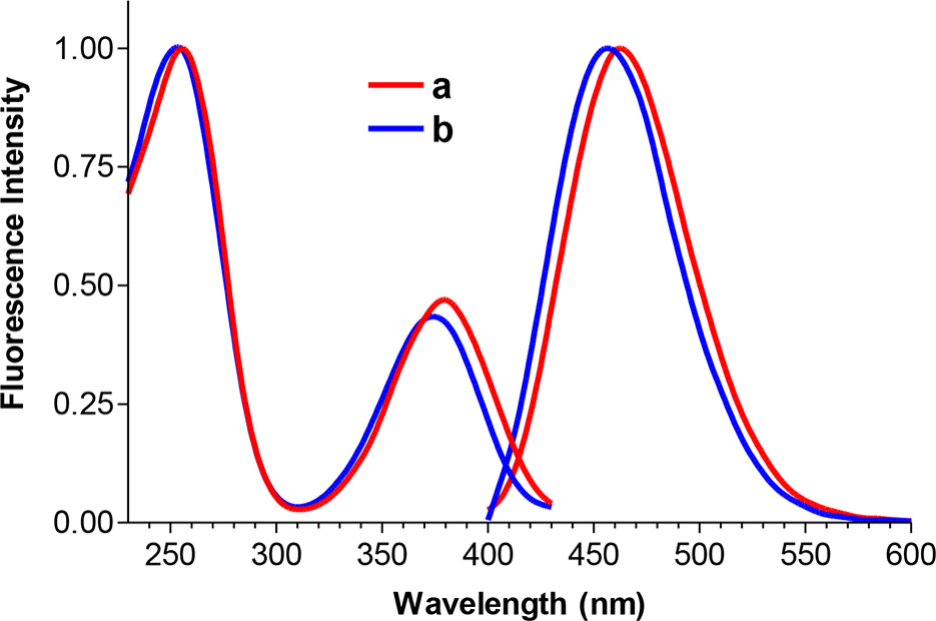
Normalized excitation (λ_em_ = 460 nm) and emission (λ_ex_ = 370 nm) spectra of photo-crosslinks **1a, 1b, 2a, 2b, 3a**, and **3b**.

Photoproducts **1a**, **1b**, **2a**, **2b**, **3a**, and **3b** have the same absorption and emission spectra (Figures 3 and 4, respectively) characteristic for the previously observed fluorescent photoadduct of ^**FS**^**U** with **T**,^15^ which were identified as two diastereomeric (**a**, **b**) intrastrand photo-crosslinks of ^**FS**^**U** with **T** (Figure 3). Fluorescent photoproducts **1a** and **1b** were the main products for **ODN 1** (Figure 2C), while only trace amounts of photoproducts **2a** and **2b** were formed for **ODN 2** (Figure 2D).

**Figure 5:**
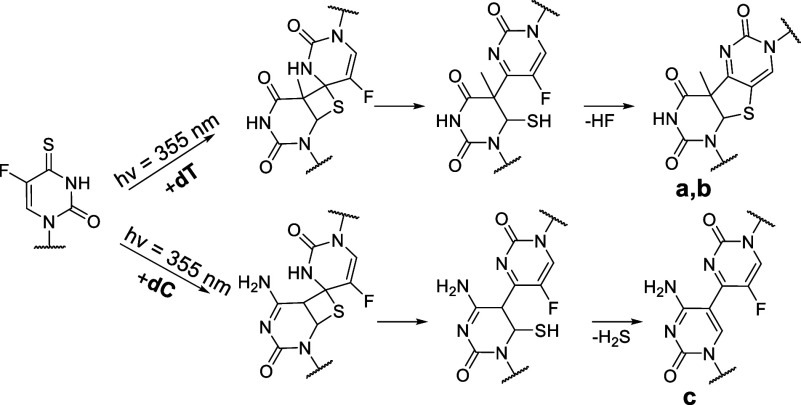
Proposed mechanism of the photo-crosslinking reaction of ^**FS**^**U** with thymine and cytosine in DNA.

The nonfluorescent photoproduct **2c** was observed in the mixture after irradiation of **ODN 2** as the dominant one (Figure 2D). A product with the same spectroscopic properties was also detected in the case of **ODN 1** (**1c**) but with a significantly lower yield. Moreover, this type of product was not observed after the irradiation of **ODN 3** (Figure 2E). In the case of **ODN 2** and **ODN 3**, photoproducts **2d** and **3d** were observed, respectively (Figure 2C,D), while no analogous photoproducts were formed in the case of **ODN 1**. In the case of **ODN 3**, the formation of photoproducts with retention times between 15 and 17 min was observed (Figure 2E, marked with *). Although they are found in relatively high yield after irradiation for 150 s, we noticed their total conversion to photoproducts **3a**, **3b**, and **3d** after irradiation of **ODN 3** for 10 min (Figure 2B). The absorption spectra of these intermediate photoproducts are presented in Figure S4. The starting **ODN 3** has an absorption maximum at 340 nm (Figure S4A), while in the case of photoproducts, this band is definitely less intense (Figure S4C,D) or shifted to the shorter wavelengths (Figure S4B). Numerous studies have shown that the formation of pyrimidine–pyrimidone photoadducts involves 2 + 2 photo-cycloaddition of C=S to C5=C6 of another pyrimidine, leading to the formation of a thermally unstable thietane in the first step.^14,17^ We proposed that this process in the case of the reaction of 5-fluoro or 5-chloro-4-thiouridine with **T** is followed by thietane ring opening, leaving the thiol on the C6 of the pyrimidine part (Figure 5).

We were able to observe thietane experimentally for duplexes labeled with 5-chloro-2′-deoxy-4-thiouridine.^13^ Taking into account the photochemical instability of the observed photoproducts after a short irradiation of **ODN 3** and their absorption properties, we strongly believe that these are isomers of the postulated intermediates.

The main photoproducts after the irradiation of **ODN 1**–**3** were isolated and separated by HPLC, and the MALDI-TOF MS spectra (Table 1, Figures S5 and S11) were recorded.

**Table 1:** MALDI-TOF MS Spectra of Isolated Photoproducts

photoproduct	calcd [M + H]^+^	found
**1a**	3065.529	3066.230
**1b**	3065.529	3065.531
**2c**	3051.547	3051.920
**3d**	3069.558	3069.326

The photoproducts **2d** and **3d** exhibit the UV absorption maximum at 260 nm, and the absence of the band with a maximum at 340 nm, characteristic of a thiocarbonyl group, is observed (Figure 3). In the MALDI-TOF MS spectrum of product **3d** (Figures S10 and S11), a signal corresponding to a mass reduced by 16 compared to **ODN 3** is present. This prompts us to claim that **3d** and **2d** are the products of the photooxidation of ^**FS**^**U** to 5-fluorouridine (**d**) (Figure 3). Additionally, we irradiated **ODN 3** under anaerobic conditions and noticed a decreased yield in the formation of photoproduct **3d** (for HPLC analysis, see Figure S12), confirming the participation of oxygen in the formation of this product.

To identify photoproduct **2c**, an additional experiment was performed by irradiating 5-fluoro-4-thiouridine in the presence of cytidine in 0.1 M phosphate buffer, pH 7.0 (see Figure S13 for the HPLC analysis after the irradiation of 5-fluoro-4-thiouridine with **C**). A triple molar equivalent of cytidine was used to minimize the formation of photoadducts of 5-fluoro-4-thiouridine itself.^18^ Comparing the absorption spectra of the products obtained after irradiation of the mixture of 5-fluoro-4-thiouridine with **C**, a similarity of the UV spectrum of one of the products (Figure S13, RT = 13.7 min) to the spectrum of photoproduct **2c** formed after irradiation of **ODN 2** was noticed (Figure S14). This product was isolated from the reaction mixture, and based on the result obtained from its ESI-MS spectrum (Figure S15) and the MALDI-TOF MS spectrum of photoproduct **2c** (Figures S7 and S8), we suggest that in the case of **ODN 2**, the intrastrand photo-crosslink with **C** (**c**) is formed. The photoadduct formation of ^**4S**^**U** with **C** observed in the *E. coli* tRNA is well described.^14,17^ We propose an analogous pathway for the photoaddition of ^**FS**^**U** to **C** with the elimination of H_2_S and the formation of the nonfluorescent 4–5 photoadduct **c** (Figure 5).

For further characterization of photo-crosslinks, we conducted enzymatic digestion of the selected photoproducts using snake venom phosphodiesterase (SVPD) and alkaline phosphatase (AP). HPLC analysis of the resulting mixtures after digestion of the photo-crosslink of ^**FS**^**U** with **T** (**3a** and **3b**) and **C** (**2c**) (Figure S16) revealed peaks corresponding to an incompletely digested fragment containing crosslinks, consistent with the enzymatic degradation pathway previously described.^12^ Moreover, the absence of the peak corresponding to **T** (in the mixture after digestion of **3a** and **3b**) and the reduced area under the peak corresponding to deoxycytidine (for **2c**) confirm the involvement of these nucleobases in the photo-crosslinking reaction with ^**FS**^**U**.

**Figure 6:**
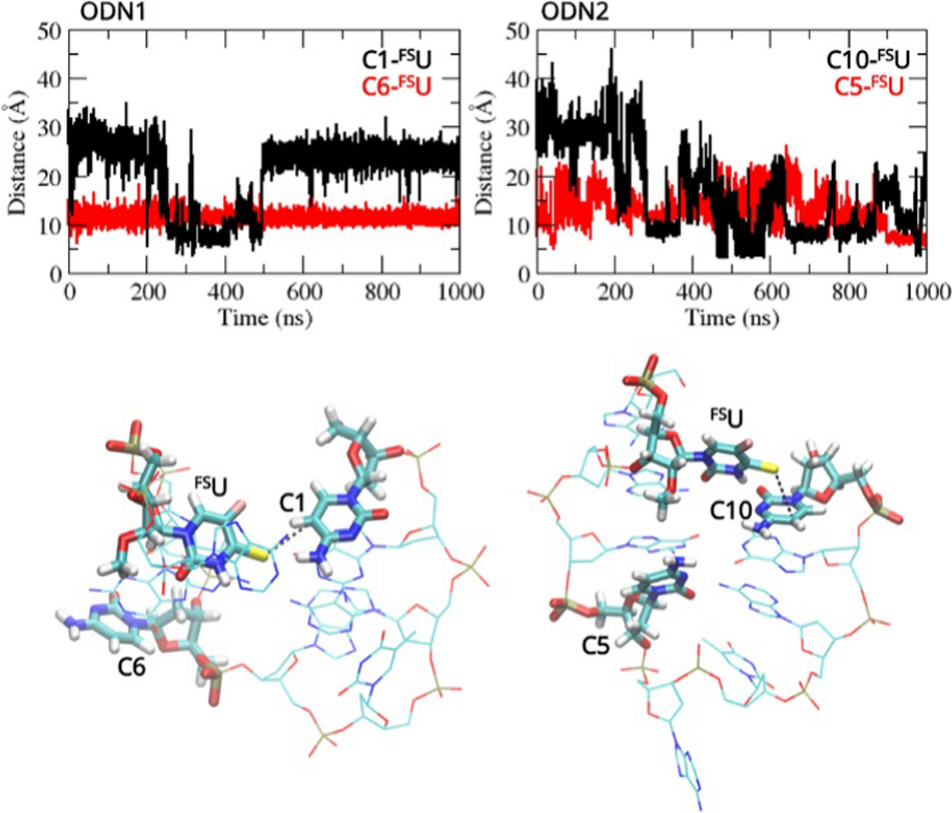
Measured distances between the thiocarbonyl group of the ^**FS**^**U** and the C=C double bond of cytosines of **ODN 1** and **ODN 2** with representative structures of **ODN 1** and **ODN 2** investigated using MD simulations.

Since short single-stranded oligonucleotides show very high flexibility, it is difficult to unequivocally determine from the obtained results which of the cytosines C1 or C6 and C5 or C10 for **ODN 1** and **ODN 2**, respectively, participates in the photo-crosslinking reaction with ^**FS**^**U**. To assess the conformational flexibility of **ODN 1** and **ODN 2**, explicit-solvent molecular dynamics (MD) simulations were performed. The simulations helped us investigate the possibility of an interaction between ^**FS**^**U** and cytosines present in the tested oligonucleotides. For both oligonucleotide systems, the MD simulations were run for 1000 ns, and distances between the thiocarbonyl group of the ^**FS**^**U** and the cytosine C=C double bond were measured. The simulations showed that cytosines located at the ends of the tested oligonucleotides are able to come into closer contact with ^**FS**^**U** compared to the cytosine in the middle of the sequence (Figure 6) and most likely they react with ^**FS**^**U** to form the nonfluorescent adduct **c** (Figure 3).

Since temperature is one of the factors affecting the conformational flexibility of single-stranded DNA molecules, we irradiated **ODN 1**–**3** under different conditions (at 5 and 35 °C). HPLC analyses of these experiments are displayed in Figure 2. Different conversions of the starting ODN was observed (Figure S17) after the same irradiation time (150 s). For all oligonucleotides, an increase in conversion was observed with increasing irradiation temperature. The highest conversion value was obtained at 35 °C (95%) for **ODN 1** but at a lower temperature of 5 °C for **ODN 2** (86%). At room temperature, the highest reactivity was also observed for **ODN 1**. In the case of **ODN 3**, a clear difference in reactivity was observed depending on the temperature (from 50% at 5 °C to 93% at 35 °C). Taking into account the reactivity of ^**FS**^**U** toward intrastrand crosslink formation with **T**, the highest selectivity was noticed in the case of irradiation of **ODN 1** at 35 °C. Also for other oligonucleotides, an increase in temperature resulted in an increase in the photo-crosslinking with **T**, while lowering the irradiation temperature favored the photo-crosslinking reaction of ^**FS**^**U** with **C**. In the case of **ODN 3**, competition between the photo-crosslinking reaction of ^**FS**^**U** with **T** and the photooxidation of ^**FS**^**U** was observed. In this case, the increase in temperature also caused an increase in the photo-crosslinking yield.

Considering the reactivity of **ODN 1**–**3** in intra- and interstrand photo-crosslinking,^12^ we concluded that the location of ^**FS**^**U** near the 3′ end of the modified strand has an advantage in the context of the application for the fluorescence-based detection of specific DNA sequences. Consequently, we designed and synthesized the ^**FS**^**U probe** oligonucleotide (Table 2) (see Figure S18 for the MALDI-TOF MS spectrum and Figure S19 for HPLC analysis), with the sequence complementary to the fragment of the E6 gene of human papillomavirus (Alphapapillomavirus 9) (HPV-16)^19^ (**target 1**). Since fluorescent photo-crosslinking is formed exclusively in the reaction of ^**FS**^**U** with **T**, all thymidines in the ^**FS**^**U probe** were replaced with deoxyuridine (**dU**) to ensure that the fluorescence signal is generated only during the interstrand photo-crosslinking of the ^**FS**^**U probe** with **T** from the target DNA.

**Table 2:** Sequences of Oligonucleotide the ^**FS**^**U Probe** and Synthetic Targets^a^

oligonucleotide	sequence 5′- 3′
^**FS**^**U probe**	GCdUCdUGdUGCA^**FS**^**U**A
**target 1 (HPV-16)**	TTATGCACAGAGC
**target 2**	TTAGTATAGTGAG
**target 3**	TTATGCGTGAGAT
**target 1_A1**	ATATGCACAGAGC
**target 1_C1**	CTATGCACAGAGC
**target 1_G1**	GTATGCACAGAGC
**target 1_G2**	TGATGCACAGAGC
**target 1_G3**	TTGTGCACAGAGC

a The underlined base indicates the base mismatch.

The oligonucleotide ^**FS**^**U probe** (10 mM in 0.1 M phosphate buffer, pH 7.0) was irradiated with 355 nm light in the presence of specific targets (1.2 molar equiv) for 5 min at room temperature. The fluorescence was measured before and after irradiation.

**Figure 7:**
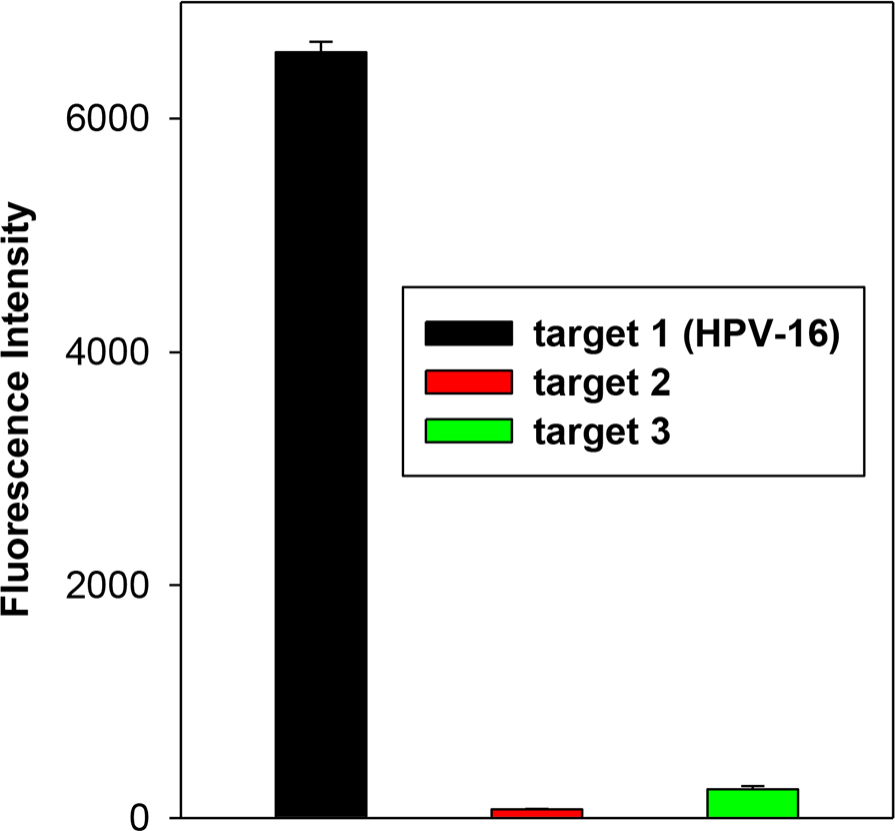
Fluorescence intensity (*F* – *F*_0_) at 450 nm (λ_ex_ = 370 nm) after irradiation of the ^**FS**^**U probe** in the presence of **targets1–3**.

Figure 7 shows that the intense fluorescence signal was generated only when the ^**FS**^**U probe** was irradiated with **target 1**. This indicates that the ^**FS**^**U probe** is selective and efficiently forms a photo-crosslinking product with **T** only in the presence of fully complementary **target 1**. The fluorescence quantum yield of the interstrand photo-crosslink of ^**FS**^**U probe** with **target 1** (Φ_f_ = 0.18) was determined relative to quinine sulfate as a reference standard.

**Figure 8:**
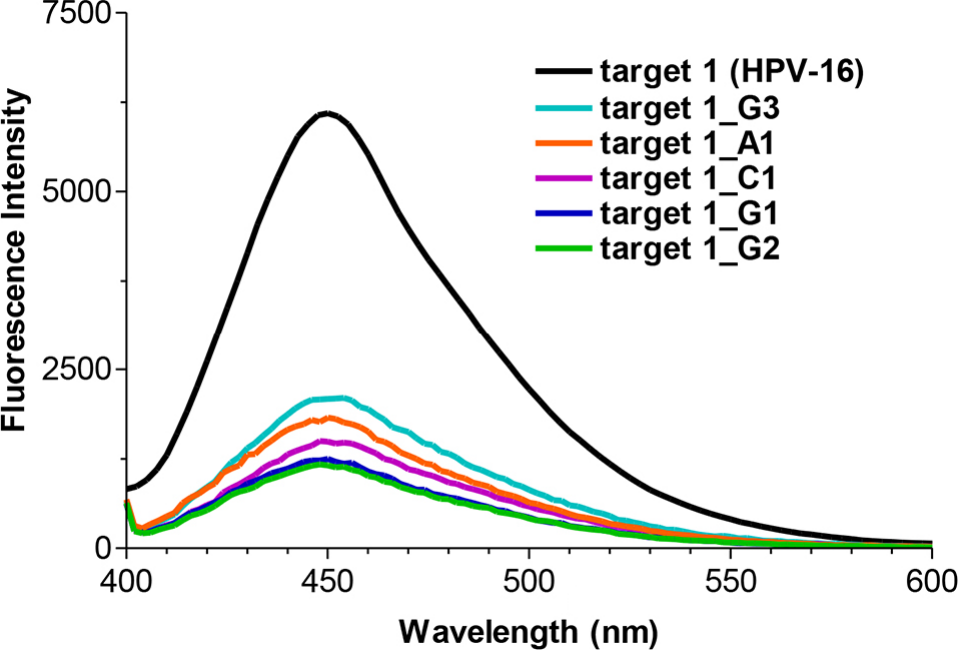
Fluorescence spectra (λ_ex_ = 370 nm) after irradiation of the ^**FS**^**U probe** in the presence of targets with a single mismatch.

We also tested the specificity of the ^**FS**^**U probe** in a photo-crosslinking reaction with synthetic targets having a single mismatch (Table 2). The fluorescence intensity was affected by the replacement of the overhanging **T1** by all bases (**A**, **C**, **G**), resulting in a significant decrease in the signal compared to the oligonucleotide **target 1** (Figure 8). Additionally, we observed the same fluorescence intensity for the oligonucleotide with a mismatch at the second position of **target 1** (**target 1_G2**), while for **target 1_G3**, the intensity was higher but still significantly reduced compared to that of **target 1**.

## Conclusions

The results showed that the intrastrand photo-crosslinking reaction can occur in short DNA fragments between nonadjacent bases. Regardless of the fact that ^**FS**^**U** shows a much higher reactivity toward thymine than cytosine, the factor determining the direction of the intrastrand photo-crosslinking reaction in short single-stranded DNA oligonucleotides is their sequence and the position of ^**FS**^**U** in the chain. For **ODN 1**, when ^**FS**^**U** is located near the 3′ end, the reaction of ^**FS**^**U** with **T** is observed, while for the reversed sequence (**ODN 2**), the photo-crosslink with **C** is formed. However, only in the case of the reaction of ^**FS**^**U** with **T**, the photo-crosslinking product is fluorescent. The location of ^**FS**^**U** closer to the middle of the strand, as in the case of **ODN 3**, results in the limitation of conformational flexibility, which makes ^**FS**^**U** difficult to arrange properly for cycloaddition to the thymidine from the complementary strand (**ODN 3**:**ODN 4** duplex). Moreover, this limitation is also reflected in the appearance of photooxidation as a competitive reaction to intrastrand photo-crosslinking. Although intrastrand reactions were observed for ^**FS**^**U**-labeled oligonucleotides, the interstrand photo-crosslinking of ^**FS**^**U** with **T** is almost quantitative when ^**FS**^**U** is located near the 3′ end.

The obtained results have great significance for designing the ^**FS**^**U**-labeled probes for the fluorescence-based detection of specific DNA sequences. Since the formation of fluorescent photo-crosslink is possible only with **T**, substituting **T** with **dU** can be considered when designing a probe with ^**FS**^**U** to avoid a false detection signal observed in the case of intrastrand photo-crosslinking.

## Experimental Section

### General Methods

HPLC was performed with an Agilent 1200 system with a binary gradient-forming module and diode array UV–vis and fluorescence detectors. Steady-state photochemical irradiation experiments were carried out in a 1 cm × 1 cm rectangular fluorescence cell with a stirring bar on a standard optical bench system equipped with a Coherent Genesis CX-355-100 cw laser and a temperature-controlled cw holder (Quantum Northwest, model TC125) and on fluorescence measurement plates. Absorption spectra were measured on a JASCO V750. Fluorescence spectra were recorded on a plate reader, TECAN Infinite M200. MS analyses were performed using the MALDI-TOF MS instrument model Autoflex II equipped with a reflectron (resolution about 5000 at *m*/*z* 1000), on a MALDI metal target plate (Bruker, Bremen, Germany). The instrument was equipped with a SmartBeam laser and operated under a FlexControl. Spectra were calibrated in FlexAnalysis using the Protein Calibration Standard I from Bruker, and 3-hydroxypicolinic acid was used as the matrix. Fluorescence excitation and emission spectra were measured at room temperature by using a JASCO Spectrofluorometer FP-8200. High-resolution electrospray ionization mass spectra (ESI-HRMS) were obtained using a Impact HD mass spectrometer (Q-TOF type instrument equipped with an electrospray ion source; Bruker Daltonics, Germany). The sample solutions (DCM:MeOH) were infused into the ESI source by a syringe pump (direct inlet) at a flow rate of 3 μL/min. The instrument was operated under the following optimized settings: end plate voltage 500 V; capillary voltage 4.2 kV; nebulizer pressure 0.3 bar; dry gas (nitrogen) temperature 200 °C; dry gas flow rate 4 L/min. The spectrometer was previously calibrated with the standard tune mixture.

### Preparation of Oligodeoxynucleotides

The synthesis of **ODN 1–4** has been previously published.^12^

The synthesis of ^**FS**^**U probe** (5′ GCdUCdUGdUGCA**FSU**A 3′) was performed on a DNA/RNA Synthesizer H-6 (K&A Laborgeraete) applying a 0.2 μmol protocol, ultramild phosphoramidites, and CPG supports according to the previously published method.^16^ The oligodeoxynucleotide ^**FS**^**U probe** was purified using reverse-phase HPLC (Waters XBridge OST C18 Column, 2.5 μm, 10 mm x 50 mm, 40 °C, with mobile phases *A* = 0.01 M CH_3_COONH_4_, *B* = 0.01 M CH_3_COONH_4_/acetonitrile, 50/50, v/v, with a diode array detector monitoring at 260 nm) using the following solvent gradient: 0–10%B in 10 min; flow rate: 1 mL/min. The HPLC fraction containing the ^**FS**^**U probe** was concentrated and desalted on an Amicon Ultra Centrifugal Filter, 3 kDa MWCO. MALDI-TOF MS (*m*/*z*): [M+H]^+^ calcd for C_115_N_42_O_72_P_11_H_132_SF 3646.2867; found 3647.9686. HPLC: 99% (RT = 11 min).

Oligonucleotide **targets 1–3** were purchased from Genomed and used without further purification.

### Irradiation and Isolation of Photo-Crosslinks

The duplex **ODN 3:ODN 4** (1: 1.1 eq) was dissolved in 0.1 M phosphate buffer (pH 7.0) to obtain a solution with *A*_260_= 2.3. The irradiation was performed at 10 °C with a Coherent Genesis CX-355–100 cw laser with 80 mW optical laser power for 10 min. Irradiations near UV light of **ODN 1–3** were carried out under aerobic conditions at 5, 20, and 35 °C in 0.1 M phosphate buffer, pH 7.0, *A*_260_= 0.5, with 80 mW optical laser power. The progress of the reaction was monitored by HPLC (analyses were performed on a Waters XBridge OST C18 Column, 2.5 μm, 4.6 mm x 50 mm, 40 °C, eluted with 0.1 M TEAA, using a linear gradient of 7–10% acetonitrile over 10 min, at a flow rate of 1 mL/min (Figure 2A,B), and on an Agilent Poroshell 120 EC-C18 Column, 2.7 μm, 4.6 mm x 150 mm, 40 °C, eluted with 0.1 M TEAA, using a linear gradient of 5–15% acetonitrile over 20 min, at a flow rate of 0.5 mL/min (Figure 2C–E)). The reaction mixture was concentrated to a small volume, and photo-crosslinks were separated using reverse-phase HPLC (Agilent Poroshell 120 EC-C18 Column, 2.7 μm, 4.6 × 150 mm, 40 °C) with the mobile phases *A* = 0.1 M CH_3_COONH_4_/acetonitrile, 95/5, v/v, *B* = 0.1 M CH_3_COONH_4_/acetonitrile, 50/50, v/v, with a diode array detector monitoring at 260 nm using the following solvent gradient: 0–15%B in 25 min; flow rate: 0.5 mL/min. The residue was evaporated and passed through a Waters XBridge OST C18 Column, 2.5 μm, 10 × 50 mm, 40 °C, with the mobile phases *A* = 0.01 M phosphate buffer (pH 7.0), *B* = acetonitrile/water, 80/20, v/v, with a diode array detector monitoring at 260 nm using a solvent gradient of 0–20%B in 12 min (flow rate: 1.5 mL/min) to remove CH_3_COONH_4_.

### Irradiation of 5-Fluoro-4-thiouridine with Cytidine

Irradiation of 5-fluoro-4-thiouridine (0.12 mM solution in 0.1 M phosphate buffer, pH 7.0) with a triple molar equivalent of cytidine was carried out under anaerobic conditions at 20 °C with 50mW optical laser power over 30 min. The product was separated by HPLC (analyses were performed on an Agilent Poroshell 120 EC-C18 Column, 2.7 μm, 4.6 mm x 150 mm, 40 °C, eluted with 0.1 M TEAA, using a linear gradient of 5–15% acetonitrile over 20 min; flow rate: 0.5 mL/min). HRMS (ESI) *m*/*z*: [M+H]^+^ calcd for C_18_H_23_FN_5_O_10_ 488.1429; found 488.1452; [M+Na]^+^ calcd for C_18_H_22_FN_5_O_10_Na 510.1248; found 510.1268.

### Irradiation of the **^FS^U Probe** with Targets

The solution of ^**FS**^**U probe** (10 μM) with oligonucleotide **targets 1–3** (12 μM) in 0.1 M phosphate buffer (pH 7.0) (100 μL) was irradiated with a Coherent Genesis CX-355–100 cw laser with 80 mW optical laser power for 5 min at room temperature on a fluorescence measurement plate. The experiment was repeated 3 times for each duplex. The fluorescence signal at 450 nm (λ_ex_ = 370 nm) was measured before and after irradiation using a plate reader TECAN Infinite M200.

### Enzymatic Digestion of Photo-Crosslinks

0.2 OD of photo-crosslink in 150 μL of buffer (10 mM KH_2_PO_4_, 10 mM MgCl_2_, pH = 7) was digested with alkaline phosphatase bovine intestinal mucosa (27 DEA units, Sigma-Aldrich, BioUltra) and phosphodiesterase I from Crotalus adamanteus venom (0.0055 units, Sigma-Aldrich, purified) for 20 h at 37 °C. The digestion mixture was analyzed by HPLC. The analysis was performed on an Agilent Poroshell 120 EC-C18 Column, (2.7 μm, 4.6 × 150 mm) at 40 °C, eluted with 0.1 M CH_3_COONH_4_, using a linear gradient of 5–6.35% acetonitrile over 5 min, followed by 11.75% acetonitrile over 5 min, followed by 50% acetonitrile over 10 min, at a flow rate of 0.7 mL/min.

### MD Simulations

The MD simulations were run in explicit solvent using ParmBsc1^20^ force field with OL15 modifications^21^ implemented in Amber20 software. Single-stranded **ODN 1** and **ODN 2** were generated using Nucgen software.^22^ The modified nucleotide was generated using ICM MolSoft program.^23^ Each system was then solvated in a cubic TIP3P waterbox whose dimensions extended to 12 Å beyond the edge of the solute atoms. The final salt concentration was maintained at 0.15 M KCl. The simulations were run in 3 steps. Step 1 consisted of 5000 steps of conjugate gradient minimization. This was followed by an equilibration step. Here, the restraints placed on the nucleotides were gradually reduced over 5 ns in an NPT ensemble. The water and ions were allowed to equilibrate with the nucleotides. The final production run consisted of 1000 ns of restraint-free simulation under the NVT ensemble with a time step of 4 fs. The simulations were run using Acemd molecular dynamics engine.^24^ The postsimulation analysis was carried out using VMD software.^25^

### Quantum Yield Measurements

The quantum yields for the disappearance of **ODN 1–3** were measured using benzophenone/benzhydrol actinometry.^26^ The fluorescence quantum yield of the photo-crosslink of ^**FS**^**U probe** with **target 1** was measured relative to quinine sulfate in 1N H_2_SO_4_ as a reference standard (Φ_f_ = 0.54).

## Data Availability

The data underlying this study are available in the published article and its Supporting Information.

## Supporting Information Available

Spectroscopic data for ^**FS**^**U probe** and all photoproducts, HPLC analysis of ^**FS**^**U probe**, conversion of **ODN 1**–**3** (%) during irradiation, HPLC analysis of **ODN 3** after irradiation under anaerobic conditions, and HPLC analyses after enzymatic digestion of photo-crosslinks (PDF)
