# Supplementary material for: Intrastrand Photo-Crosslinking of 5-Fluoro-2′-O-methyl-4-thiouridine-Modified Oligonucleotides and Its Implication for Fluorescence-Based Detection of DNA Sequences
Source: J Org Chem. 2024 Oct 24;89(23):17155–62. doi: 10.1021/acs.joc.4c01597 (PMC11629290; doi:10.1021/acs.joc.4c01597)
Supplement: Supplementary file 1 — jo4c01597_si_001.pdf [file jo4c01597_si_001.pdf]

Supporting Information

Intrastrand Photocrosslinking of 5-Fluoro-2'-O-Methyl-4-Thiouridine Modified Oligonucleotides and its Implication for Fluorescence-Based Detection of DNA Sequences

Joanna Nowak-Karnowska[1], Katarzyna Taras-Goslinska[1], Shozeb Haider[2], Bohdan Skalski[3]*

[1] Adam Mickiewicz University, Department of Chemistry, Poznań, 61-614, Poland, *email: j.nowak@amu.edu.pl

[2] University College London, School of Pharmacy, London, WC1N 1AX, United Kingdom

[3] Adam Mickiewicz University, Center for Advanced Technology, Poznań, 61-614, Poland

Table of Contents:

Figure S1. MALDI-TOF MS spectra of interstrand crosslinks of **ODN 3**: **ODN 4** S2

Figure S2. Changes in absorption spectra of **ODN 1-3** during irradiation S2

Figure S3. Conversion of **ODN 1-3** during irradiation with 355 nm at 20°C S3

Figure S4. Absorption spectra of **ODN 3** and intermediate photoproducts of irradiation of **ODN 3** S3

Figure S5. MALDI-TOF MS spectrum of photoproduct **1a** S4

Figure S6. MALDI-TOF MS spectrum of photoproduct **1b** S4

Figure S7 and S8. MALDI-TOF MS spectrum of photoproduct **2c** S5

Figure S9. MALDI-TOF MS spectra of photoproduct **3a** S6

Figure S10. MALDI-TOF MS spectra of photoproduct **3b** and **3d** S6

Figure S11. MALDI-TOF MS spectrum of photoproduct **3d** S7

Figure S12. HPLC analysis of **ODN 3** after 150 s of irradiation in aerobic and anaerobic conditions S7

Figure S13. HPLC analysis of the mixture of cytidine with 5-fluoro-4-thiouridine before and after irradiation S8

Figure S14. Absorption spectra of photocrosslink **2c** and photoadduct of irradiation of 5-fluoro-4-thiouridine with cytidine S8

Figure S15. Proposed structure and ESI-MS spectrum of photoadduct of 5-fluoro-4-thiouridine with cytidine S9

Figure S16. HPLC chromatograms after enzymatic digestion with SVPD and AP of photocrosslink **3a**, **3b** and **2c** S10

Figure S17. Conversion of **ODN1-3** (%) depending on temperature of irradiation S11

Figure S18. MALDI-TOF MS spectrum of ^{FS}**U probe** S11

Figure S19. HPLC absorbance elution profile at 260 nm of ^{FS}**U probe** S12

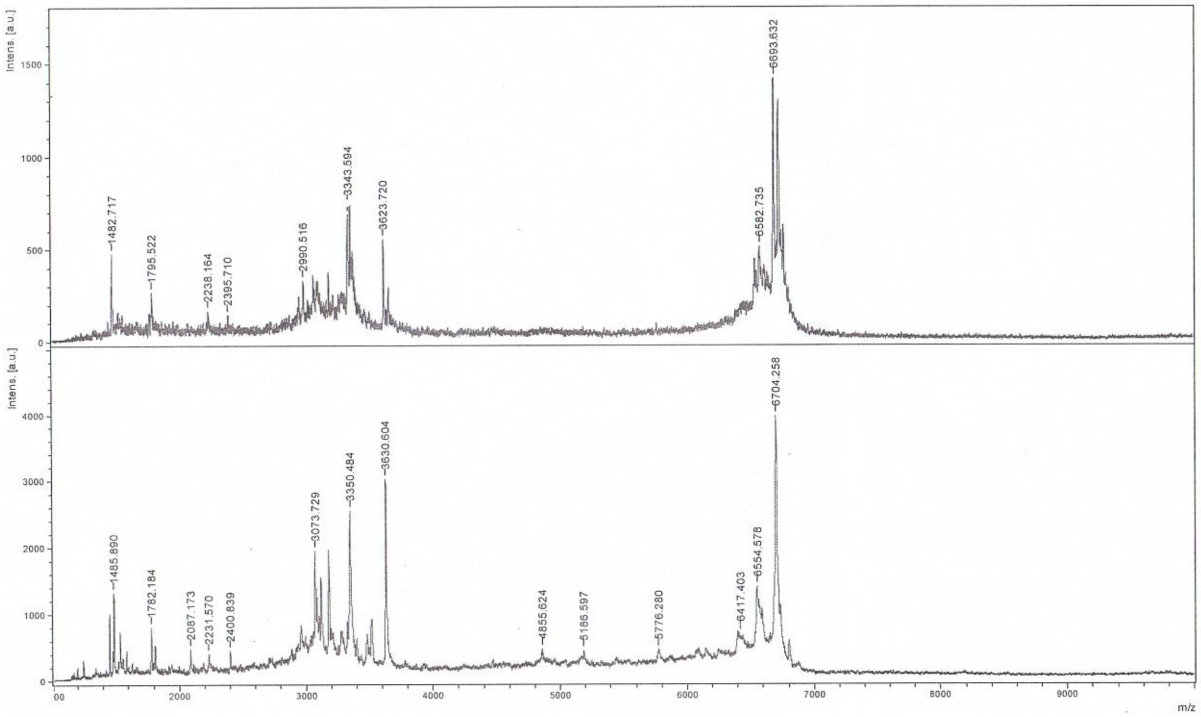

Figure S1. MALDI-TOF MS spectra of interstrand crosslinks of **ODN 3**: **ODN 4** (mass calcd 6692 for $[M+H]^+$, found 6693.632 and 6704.258).

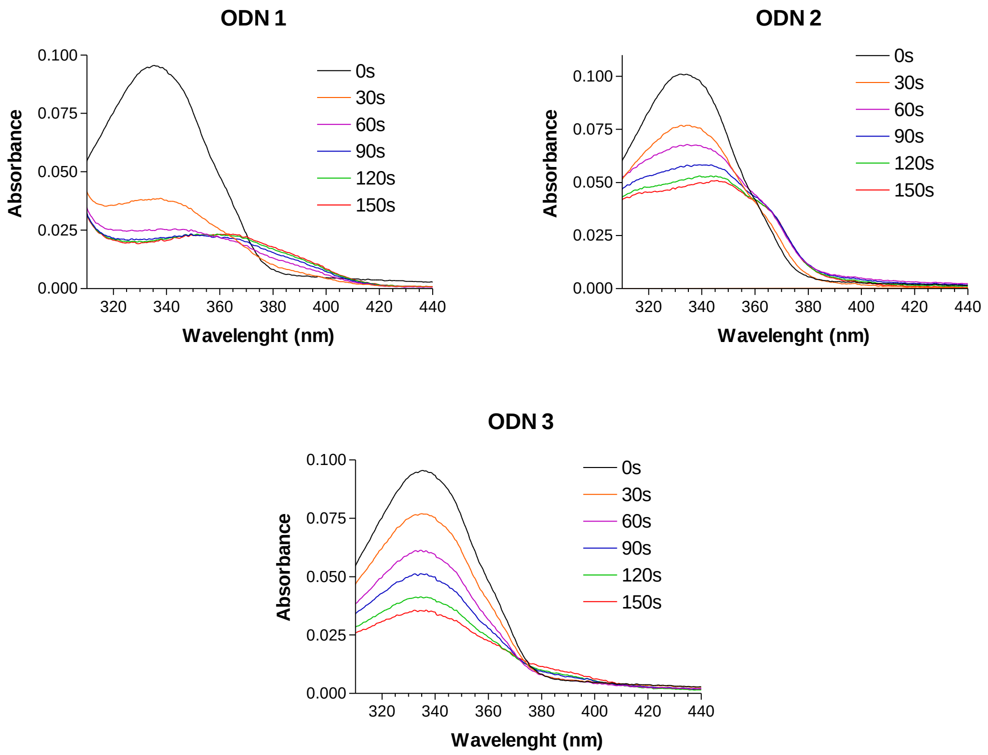

Figure S2. Changes in absorption spectra of **ODN 1-3** during irradiation with 355 nm at 20°C.

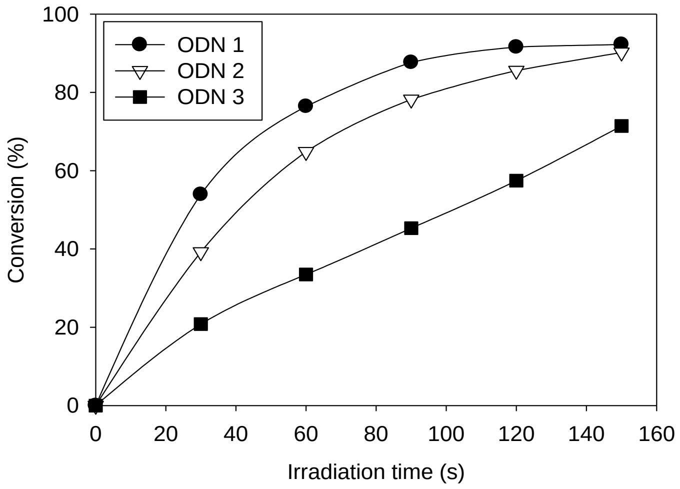

Figure S3. Conversion of **ODN 1-3** during irradiation with 355 nm at 20°C (based on HPLC signals at 260 nm).

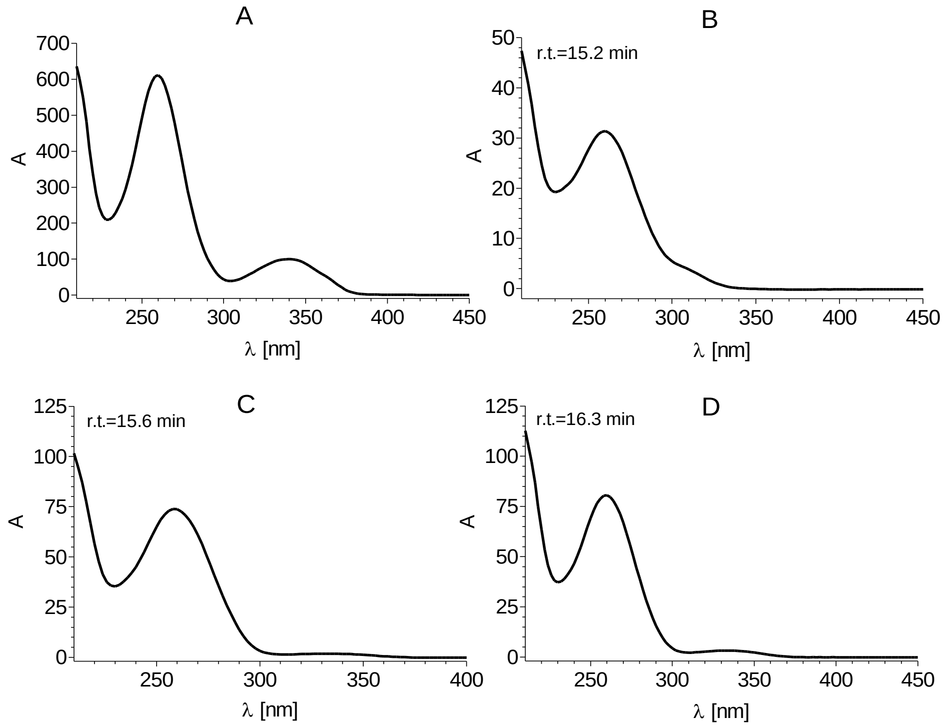

Figure S4. Absorption spectra of **ODN 3** (**A**) and intermediate photoproducts of irradiation of **ODN 3** (**B**-**D**).

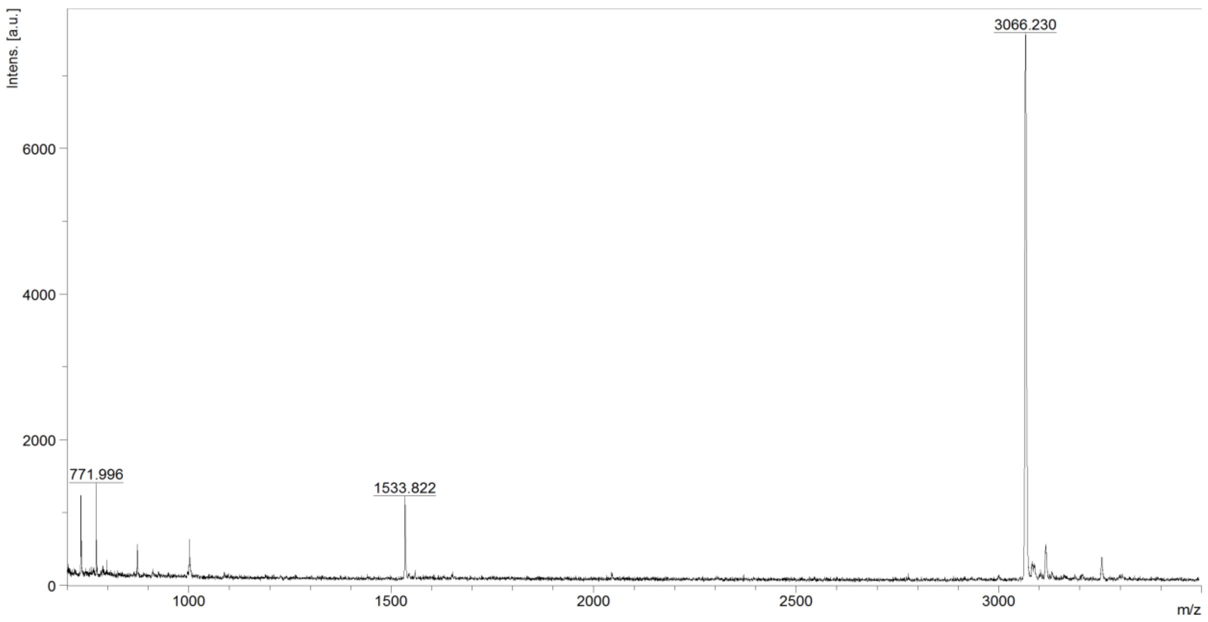

Figure S5. MALDI-TOF MS spectrum of photoproduct **1a** (mass calcd 3065.529 for $[M+H]^+$, found 3066.230).

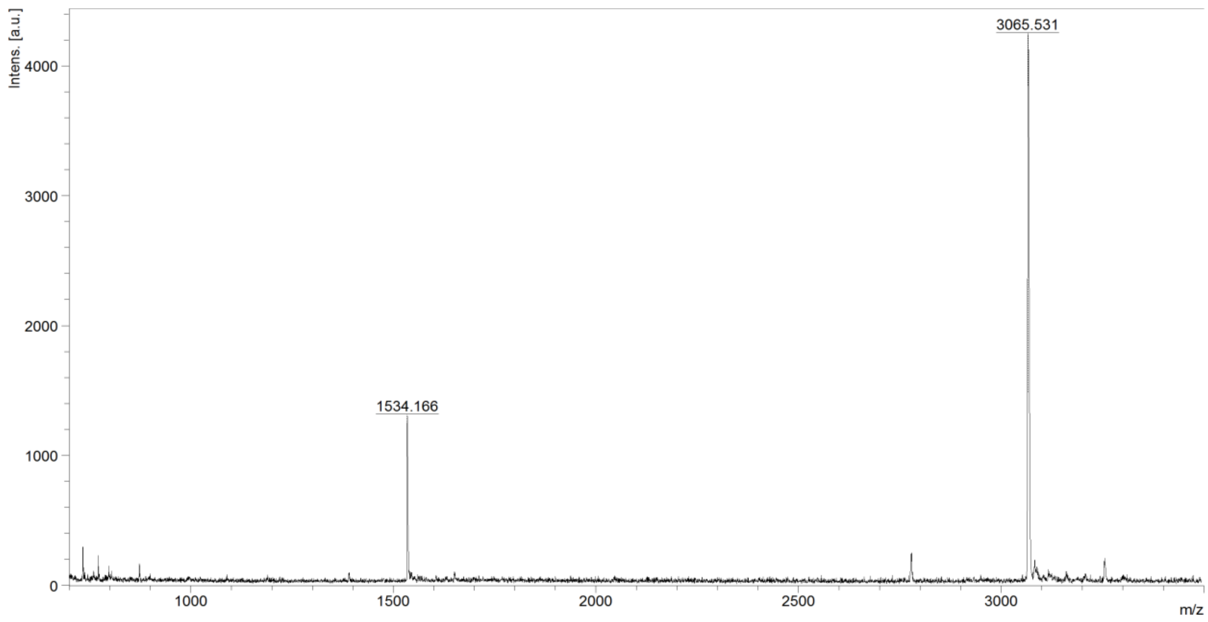

Figure S6. MALDI-TOF MS spectrum of photoproduct **1b** (mass calcd 3065.529 for $[M+H]^+$, found 3065.531).

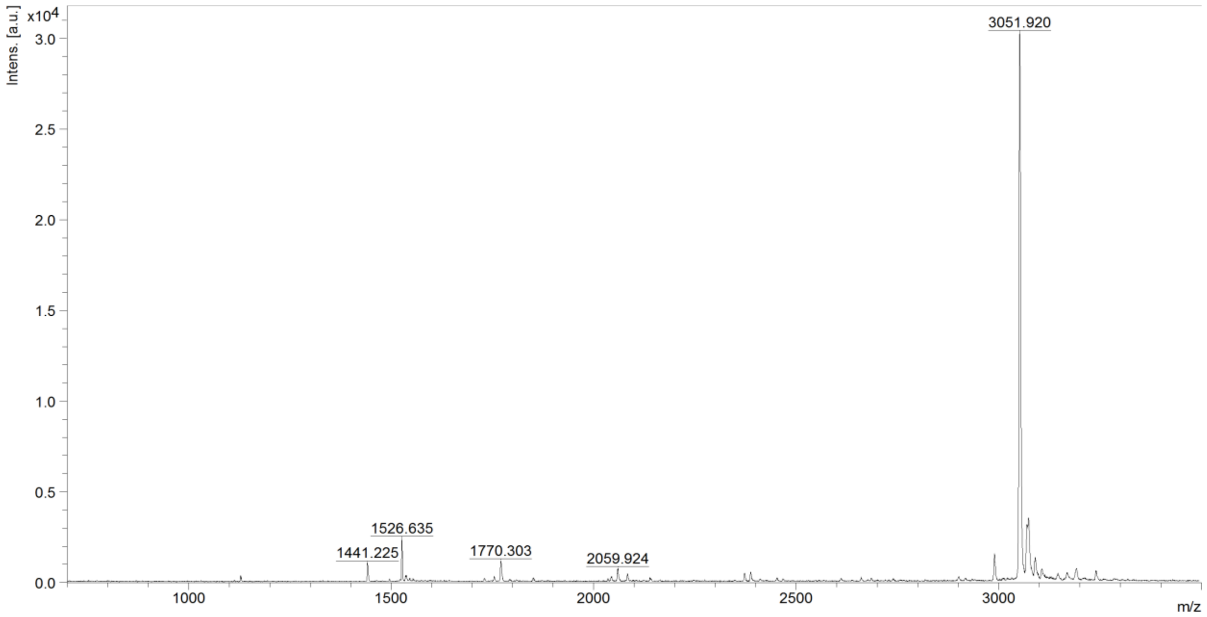

Figure S7. MALDI-TOF MS spectrum of photoproduct **2c** (mass calcd 3051,547 for $[M+H]^+$, found 3051.920).

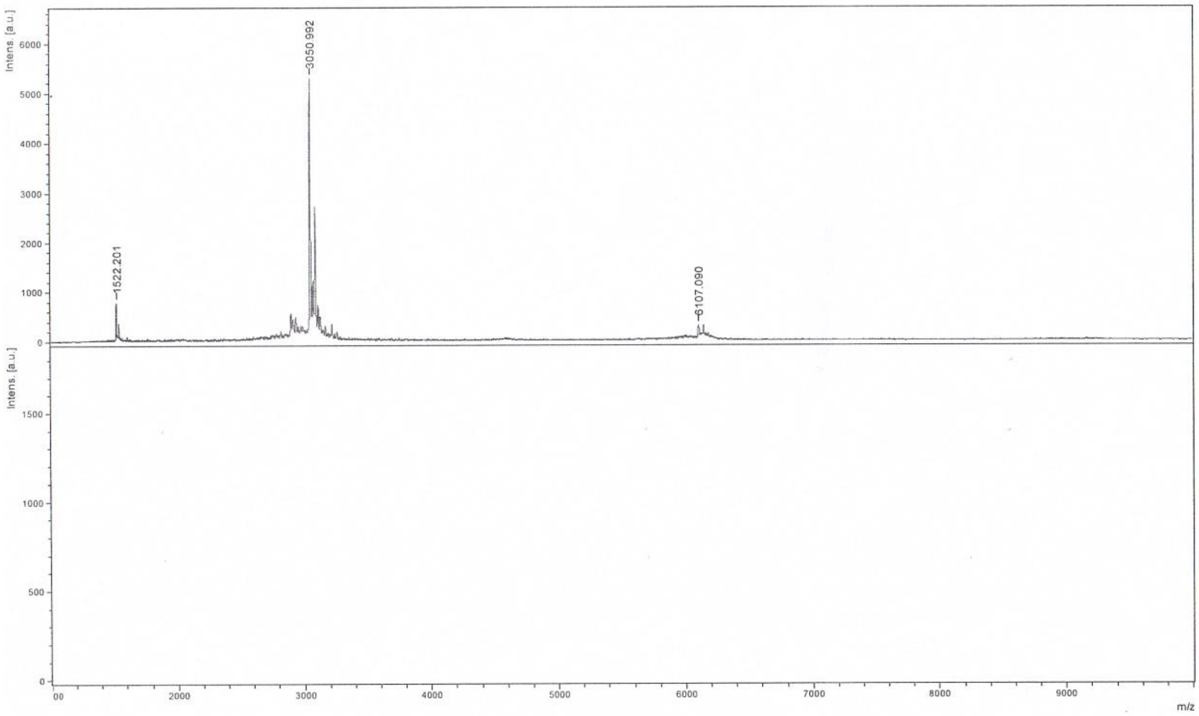

Figure S8. MALDI-TOF MS spectrum of photoproduct **2c** (mass calcd 3051,547 for $[M+H]^+$ and 6102.087 for $[2M+H]^+$, found 3050.992 and 6107.090).

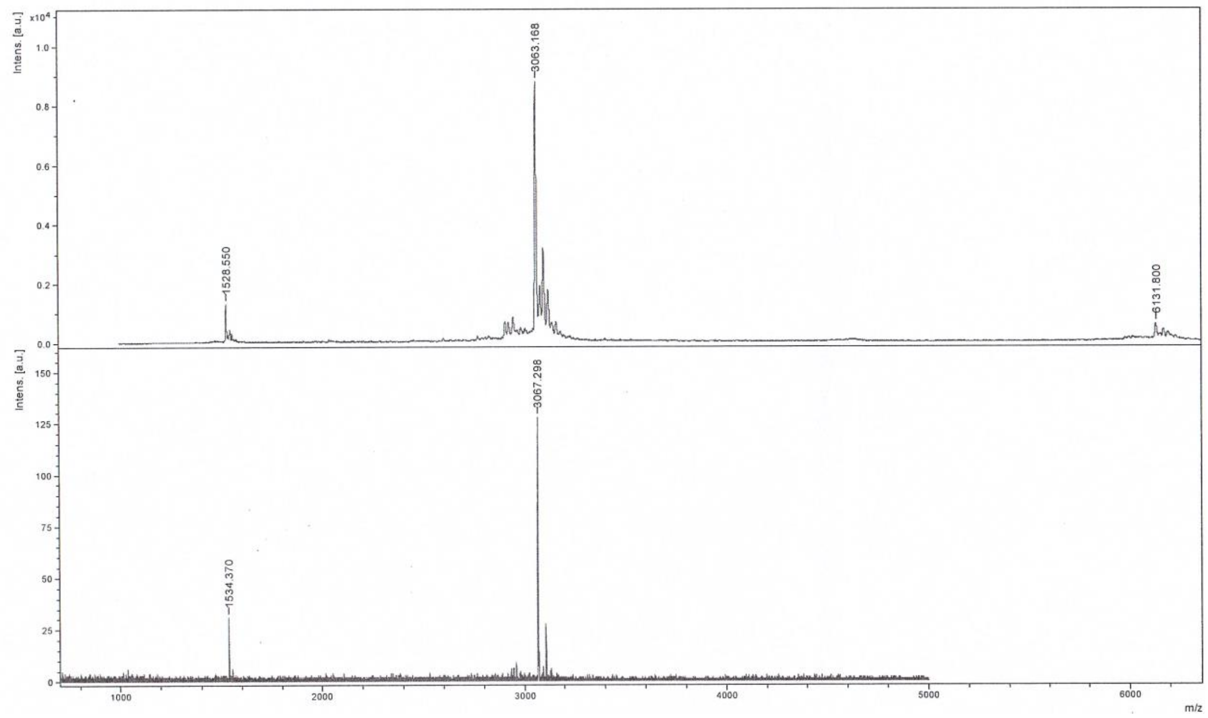

Figure S9. MALDI-TOF MS spectra of photoproduct **3a** (mass calcd 3065.529 for $[M+H]^+$ and 6130.051 for $[2M+H]^+$, found 3063.168, 3067.298 (HR, *lower part*) and 6131.800).

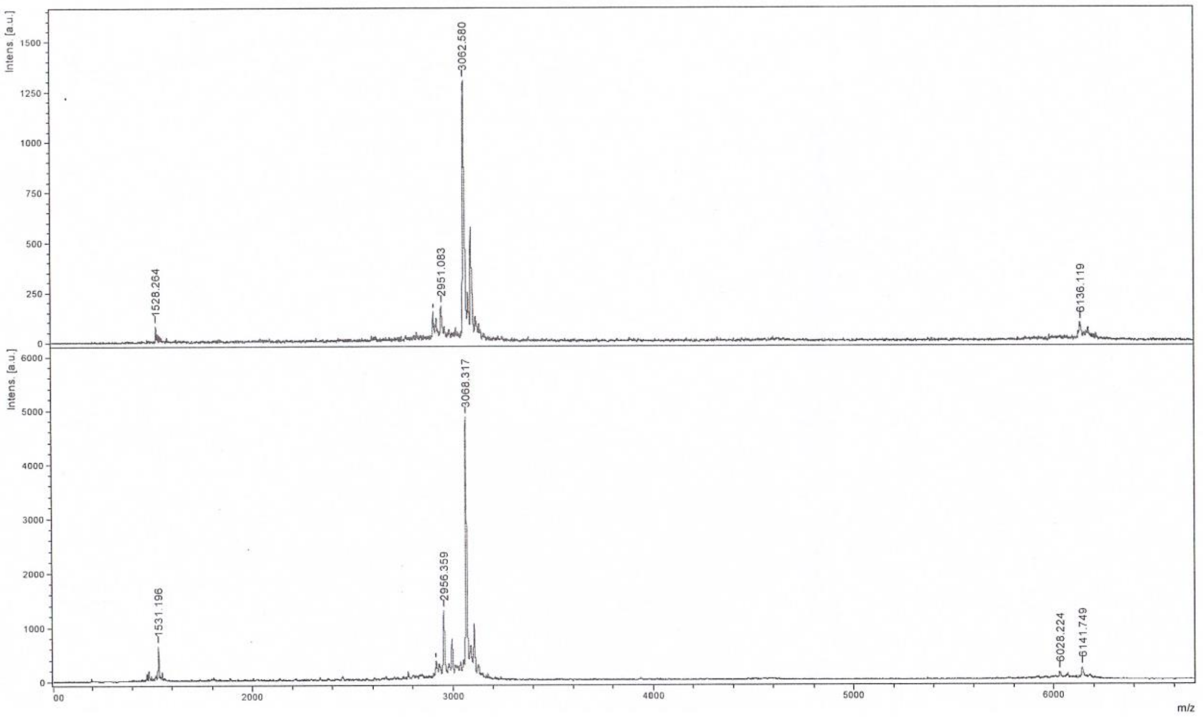

Figure S10. MALDI-TOF MS spectra of photoproduct **3b** (*upper part*)(mass calcd 3065.529 for $[M+H]^+$ and 6130.051 for $[2M+H]^+$, found 3062.580 and 6136.119) and photoproduct **3d** (*lower part*)(mass calcd 3069.558 for $[M+H]^+$and 6138.109 for $[2M+H]^+$, found 3068.317 and 6141.749).

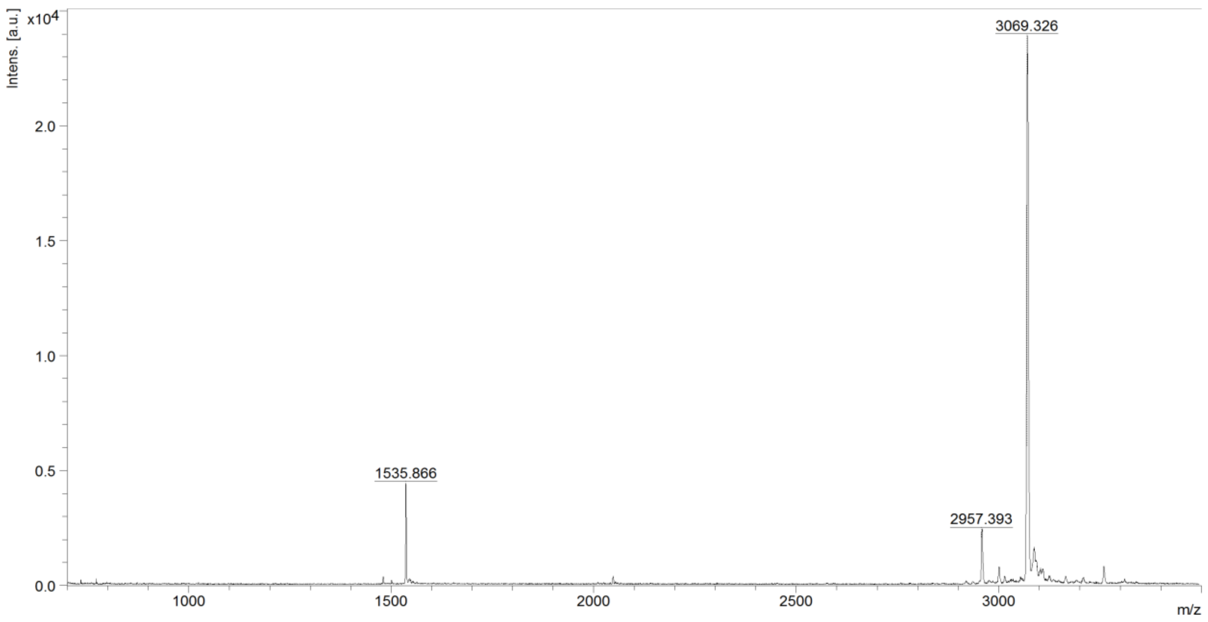

Figure S11. MALDI-TOF MS spectrum of photoproduct **3d** (mass calcd 3069.558 for $[M+H]^+$, found 3069.326).

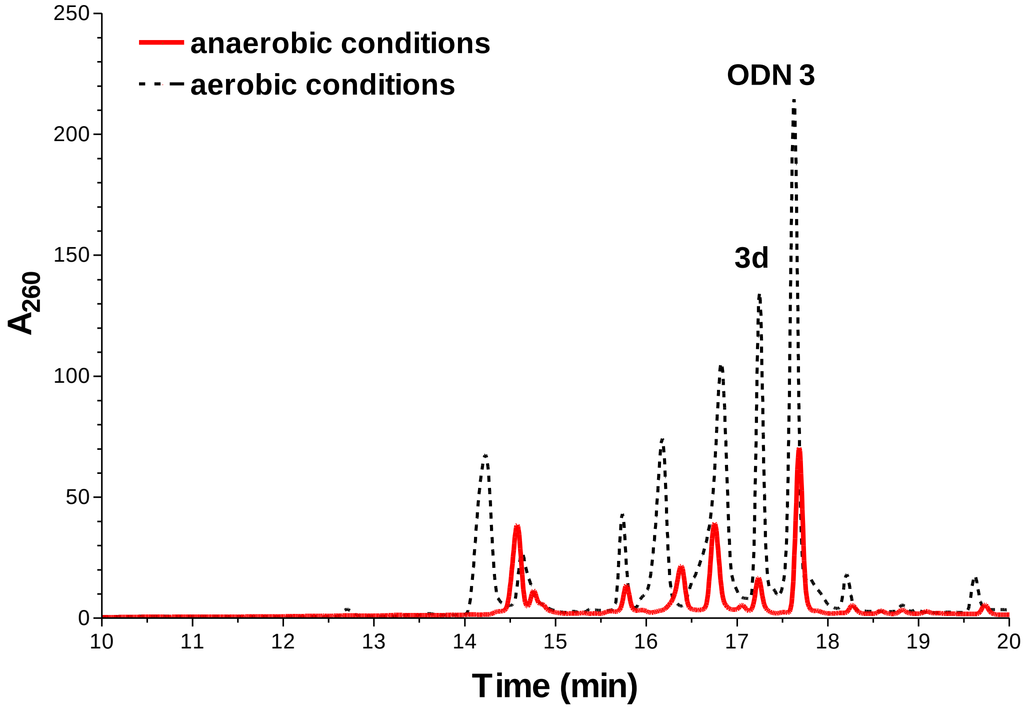

Figure S12. HPLC analysis of **ODN 3** after 150 s of irradiation with 355 nm in 20℃ in aerobic (*dotted black*) and anaerobic (*red*) conditions.

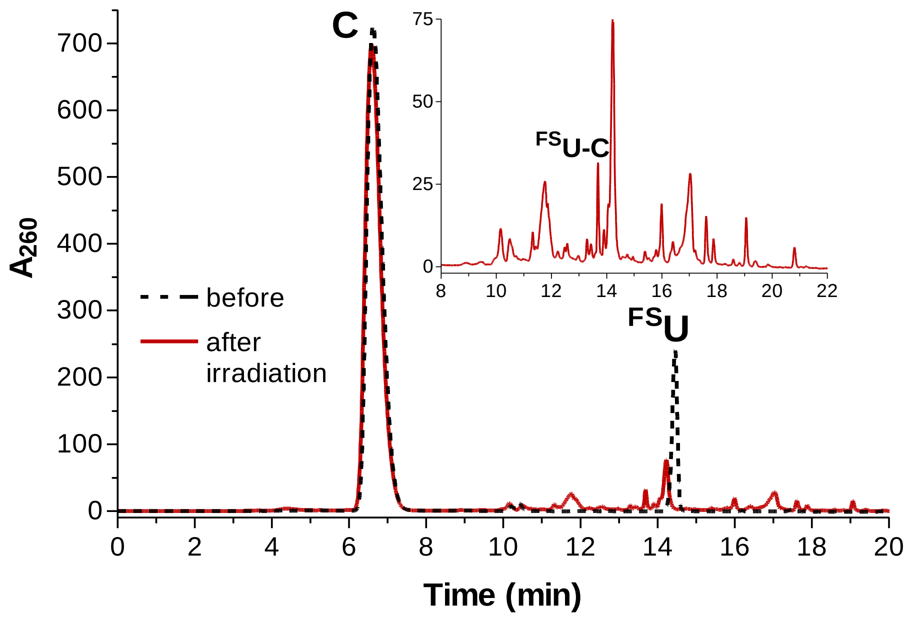

Figure S13. HPLC analysis of the mixture of cytidine with 5-fluoro-4-thiouridine before (*dotted black*) and after (*red*) irradiation. Inset shows the expanded region of the chromatogram with retention time of 8-22 min, peak marked: photoadduct (RT = 13.7 min)

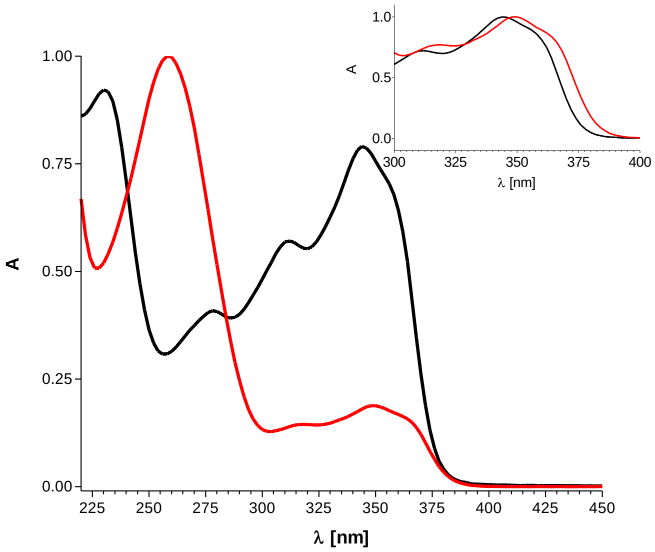

Figure S14. Absorption spectra of photocrosslink **2c** (*red*) and photoadduct of irradiation of 5-fluoro-4-thiouridine with cytidine in 0.1M phosphate buffer, pH=7 (*black*).

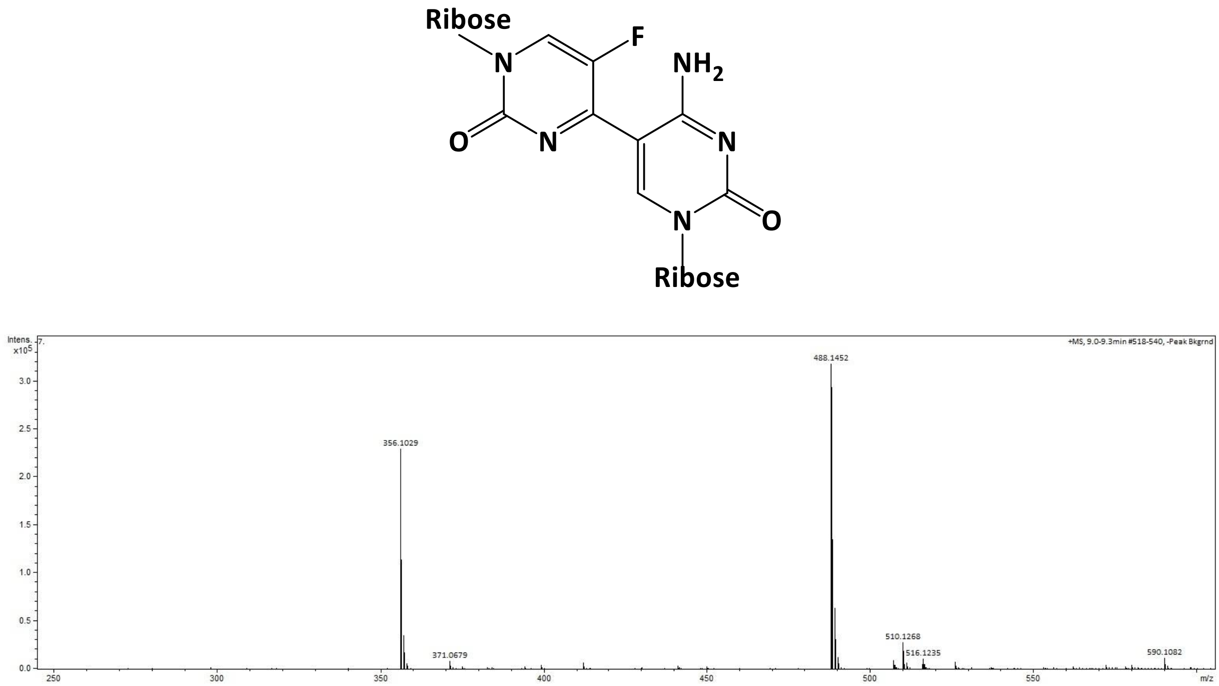

Figure S15. Proposed structure and ESI-MS spectrum of photoadduct of 5-fluoro-4-thiouridine with cytidine.

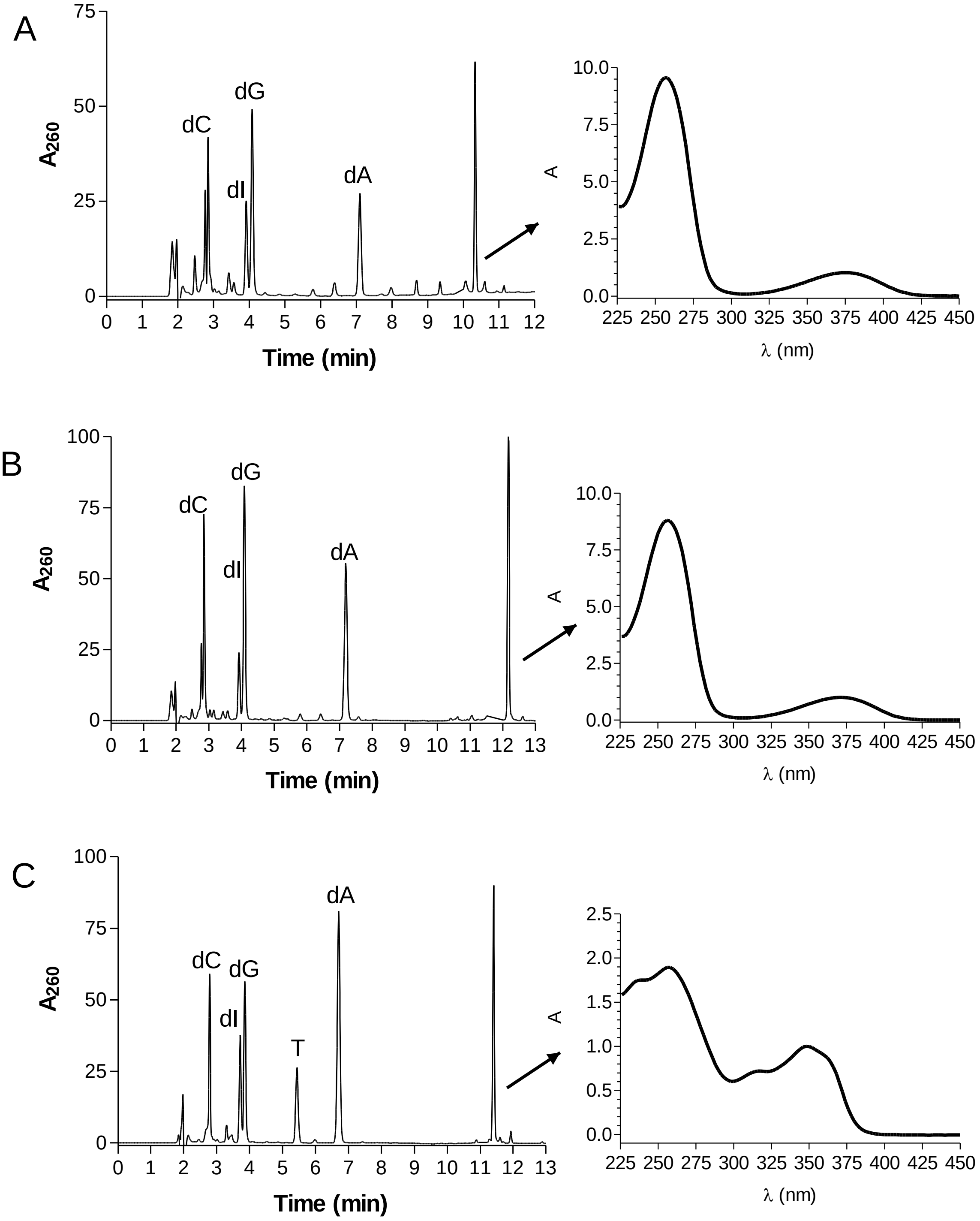

Figure S16. HPLC chromatograms (monitored at 260 nm) after enzymatic digestion with SVPD and AP of photocrosslink **3a** (**A**), **3b** (**B**) and **2c** (**C**) with UV-VIS spectra of incompletely digested fragments containing photocrosslink **FSU-T** (**A, B**) and **FSU-C** (**C**).

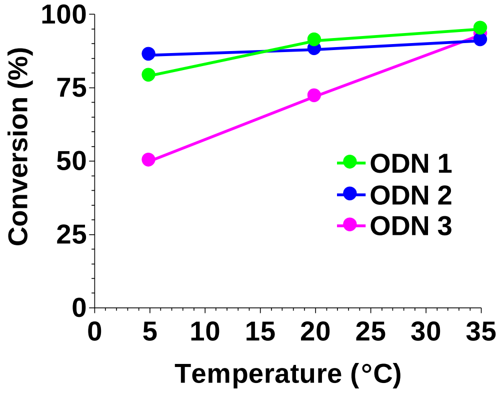

Figure S17. Conversion of **ODN 1**-**3** (%) depending on temperature of irradiation (based on HPLC data at 260 nm after 150 s of irradiation with 355 nm).

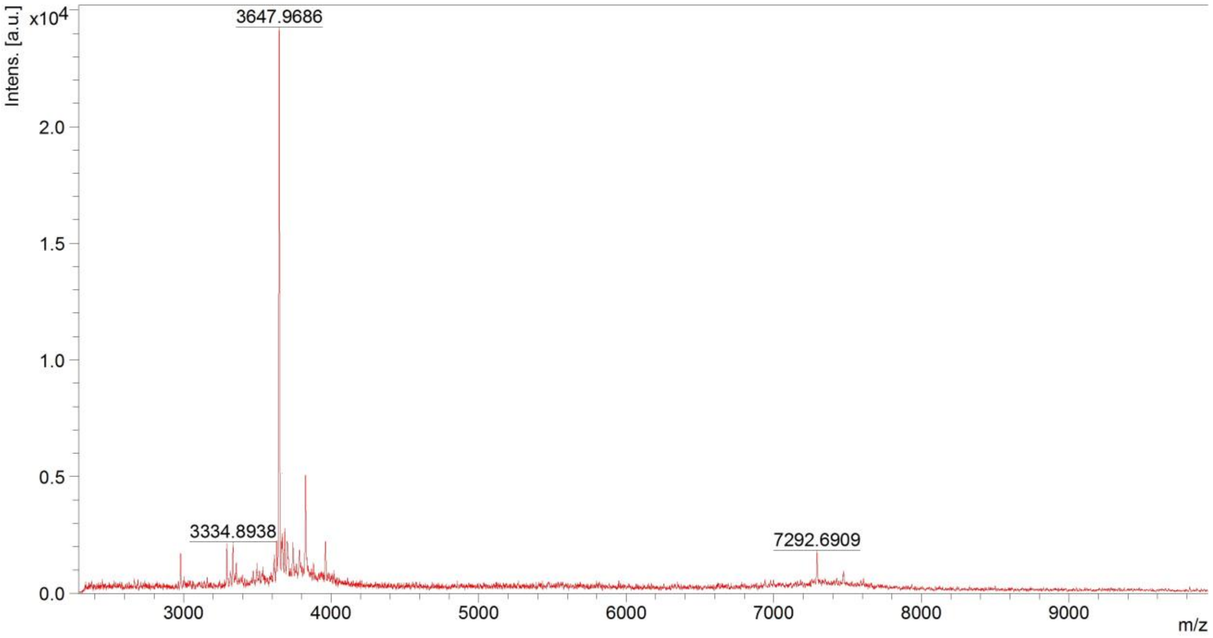

Figure S18. MALDI-TOF MS spectrum of **^{FS}U probe**.

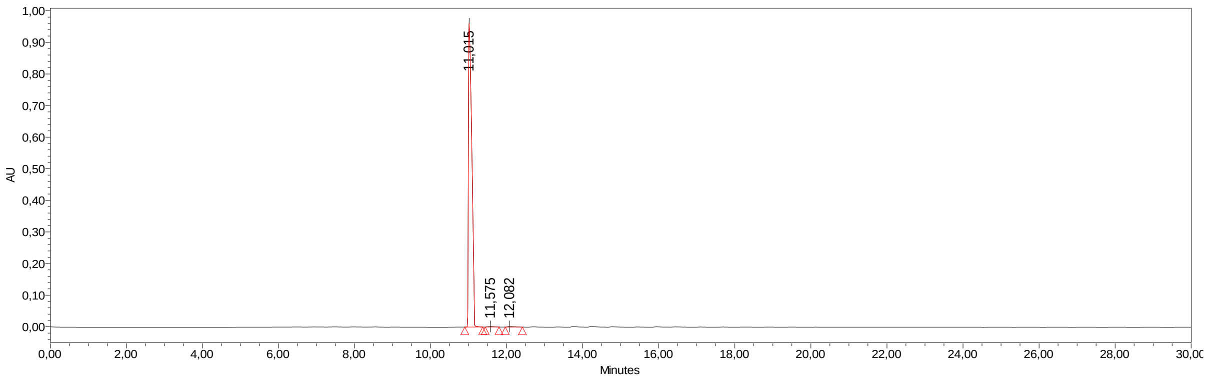

	Retention Time	Area	% Area	Height
1	11,015	5948072	99,23	961083
2	11,575	19526	0,33	1877
3	12,082	26705	0,45	2351

Figure S19. HPLC absorbance elution profile at 260 nm of **^{FS}U probe** (Agilent AdvanceBio Oligonucleotides 2.1 x 150mm, 2.7µm, 100% A to 40% A in 30 minutes, flow: 0.4 ml/min, A=0.1 M TEAA, B=0.1 M TEAA, 80% ACN).
